# Treatment of Methotrexate-Associated Lymphoproliferative Disorder With Biological Therapies

**DOI:** 10.7759/cureus.76751

**Published:** 2025-01-01

**Authors:** Kava C Afu, Andrew S Durkee

**Affiliations:** 1 Department of Internal Medicine, San Antonio Uniformed Services Health Education Consortium, San Antonio, USA; 2 Department of Hematology and Oncology, William Beaumont Army Medical Center, Fort Bliss, USA; 3 Department of Hematology and Oncology, San Antonio Uniformed Services Health Education Consortium, San Antonio, USA

**Keywords:** ebv-positive b-cell lymphoproliferative disease, hodgkin lymphoma-like disease, iatrogenic immunodeficiency-associated lymphoproliferative disorders, methotrexate-induced lymphoproliferative disorder, rituximab and brentuximab combined therapy

## Abstract

Lymphoproliferative disorders may arise as a complication of immunosuppressant medications, such as methotrexate. This case report describes a patient who developed a rare subtype of methotrexate-associated lymphoproliferative disorder. His disease initially responded well to the withdrawal of methotrexate. However, several months after diagnosis, surveillance testing revealed progressive lymphadenopathy. Owing to his multiple comorbidities and resultant poor baseline functional status, he was not a candidate for cytotoxic chemotherapy. Based on key histopathological characteristics of his rare disorder, his care team devised an alternative therapy, consisting of rituximab and brentuximab, a unique protocol that is not well-described in the lymphoma literature. The patient achieved a brief but complete remission from this therapy.

## Introduction

Individuals may develop lymphoproliferative disorders (LPDs) in the setting of immune deficiency or suppression, an association that is well-documented [1]. According to the World Health Organization classification of tumors of hematopoietic and lymphoid tissues - Revised 4th edition, LPDs were categorized according to pre-existing immunosuppressive conditions, including primary immunodeficiencies; HIV infection; post-transplantation; and other iatrogenic immunodeficiencies (OIIA). Most well-described among the OIIA is iatrogenic immunosuppression associated with methotrexate (MTX) administration [2].

MTX is an antimetabolite drug used to treat several neoplastic and rheumatologic conditions. It is most notable for its use as a first-line disease-modifying anti-rheumatic drug (DMARD) in the management of rheumatoid arthritis (RA). Lymphoma related to immunosuppression from low-dose MTX is a known but rare complication in the management of RA, with an annualized risk of 0.000003% [3]. The first report of a patient on MTX who developed LPD is from 1991 in a patient with RA [1]. Many similar reports (e.g., [2,4]) have since been published, both in patients with RA and those with other autoimmune diseases treated with MTX. The iatrogenic illness in these cases is termed methotrexate-associated LPD (MTX-LPD). The pathogenesis is not fully understood, but in some cases, the oncogenicity of the Epstein-Barr virus (EBV) is unchecked by an immune system suppressed by MTX. Due to the rarity of this side effect and the heterogeneity of MTX-LPD, no clear treatment appears to be the standard. We present a case of MTX-LPD with a rare subtype treated with a unique therapy protocol.

This article was previously presented as a poster at the 2023 Tri-Service ACP Meeting on November 9, 2023.

## Case presentation

**Figure 1:**
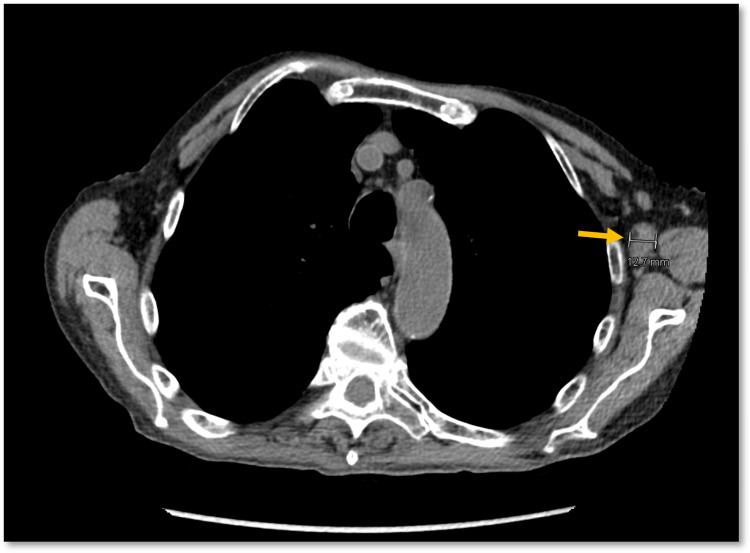
CT chest demonstrating left axillary lymphadenopathy The measurement bar (end of the yellow arrow) indicates that the largest diameter of the enlarged lymph node was 12.7 mm. CT, computed tomography

**Figure 2:**
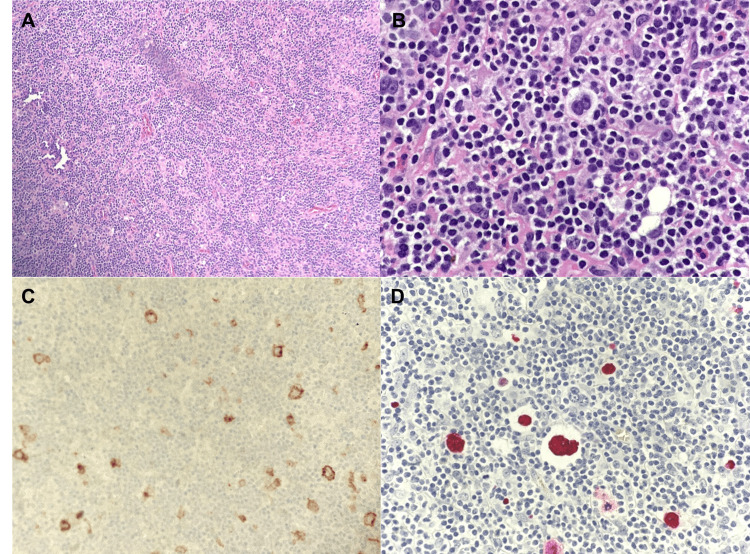
Histopathology slides from the left axillary lymph node biopsy Figure 2A presents an H&E stain of the lymph node, showing areas of normal tissue and areas of disease, including Reed-Sternberg cells, which are seen more clearly in the zoomed H&E stain, Figure 2B. Figure 2C is a CD30 stain, demonstrating the presence of Hodgkin cells. Figure 2D represents Epstein-Barr virus in ISH, showing the presence of the virus in large Hodgkin-like cells. H&E, hematoxylin and eosin; CD, cluster of differentiation; ISH, in situ hybridization

A 79-year-old man, with a history of RA, treated with MTX and adalimumab for over 20 years, presented to the emergency department after a mechanical fall. He reported rib pain and - on a CT scan ordered to rule out rib fracture - was incidentally found to have bulky left axillary lymphadenopathy (Figure 1). He had no symptoms consistent with lymphoma, but tissue sampling was pursued by his primary care manager (PCM) on follow-up. Initial pathology results were inconclusive, and the sample was sent for expert opinion, which - based on his clinical history and current medications - resulted in the diagnosis of iatrogenic EBV-positive B-cell lymphoproliferative disorder with Hodgkin-like features (Figures 2A-2D).

**Figure 3:**
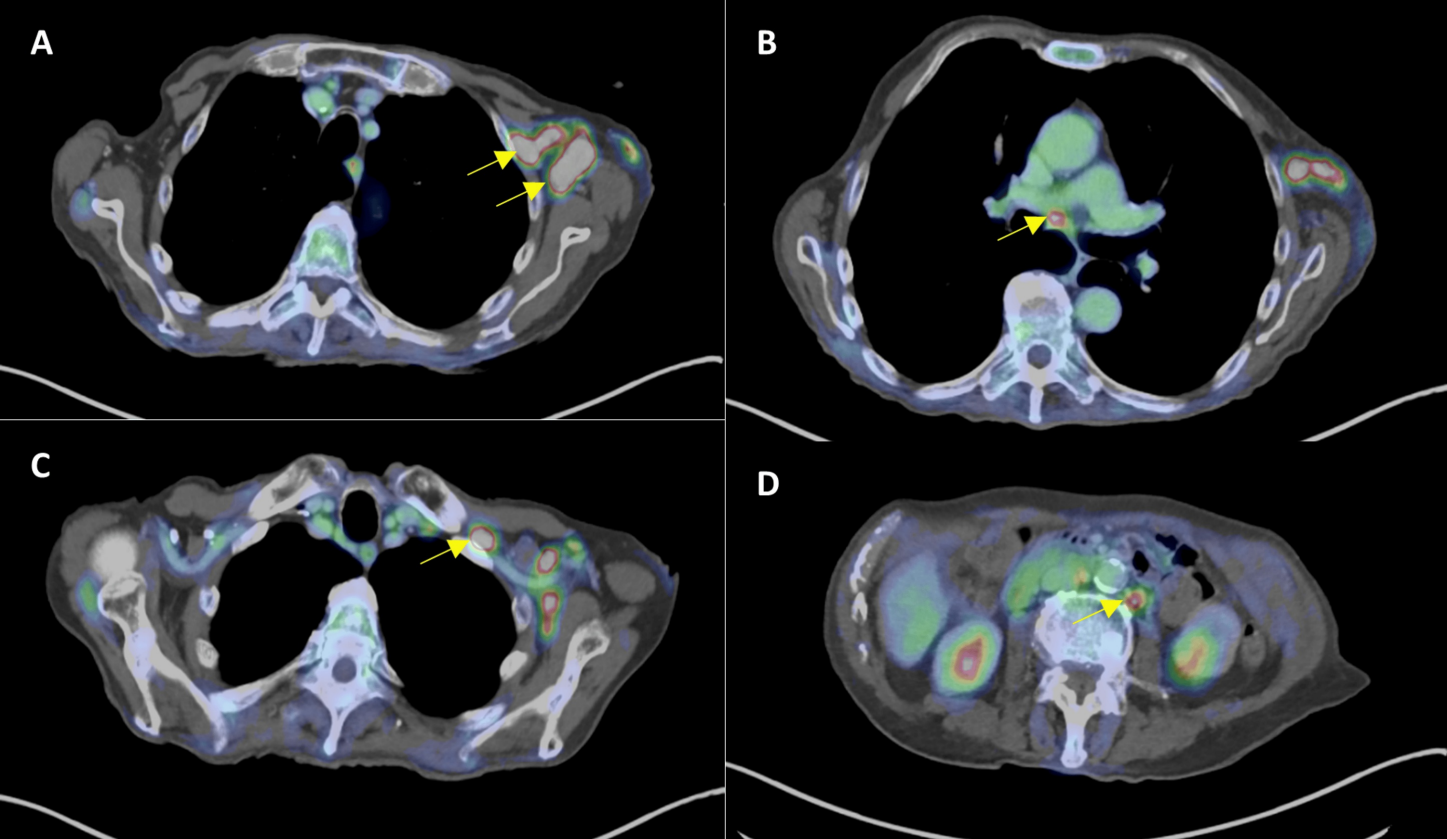
Initial PET imaging showing diffuse lymphadenopathy Figure 3A shows bulky left axillary lymph nodes, the largest measuring up to 3 x 1.9 cm. Figure 3B shows a mediastinal lymph node measuring 1.2 cm. Figure 3C shows an infraclavicular lymph node measuring 1.5 x 0.7 cm. Figure 3D shows a left para-aortic retroperitoneal lymph node measuring 0.5 cm. Respective lymph nodes are indicated by yellow arrows in each pane. PET, positron emission tomography

**Figure 4:**
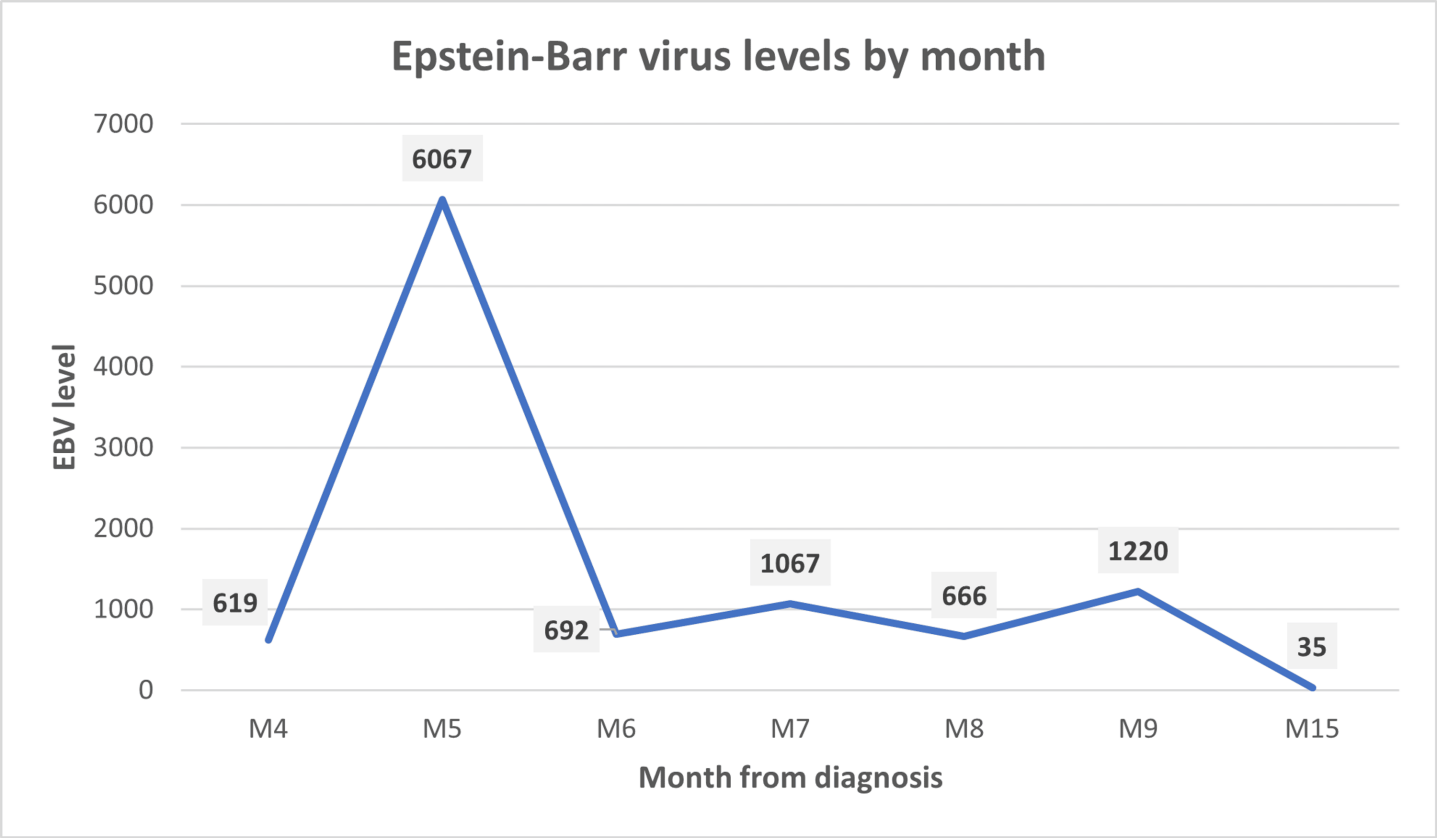
Epstein-Barr virus levels over the course of the disease EBV level trend (measured in copies/mL), M4 representing month four after diagnosis. The large reduction in the EBV level occurred after withdrawal of immunosuppression between months five and six. EBV, Epstein-Barr virus; M4, month 4 after diagnosis; M15, month 15 after diagnosis (and so on)

**Figure 5:**
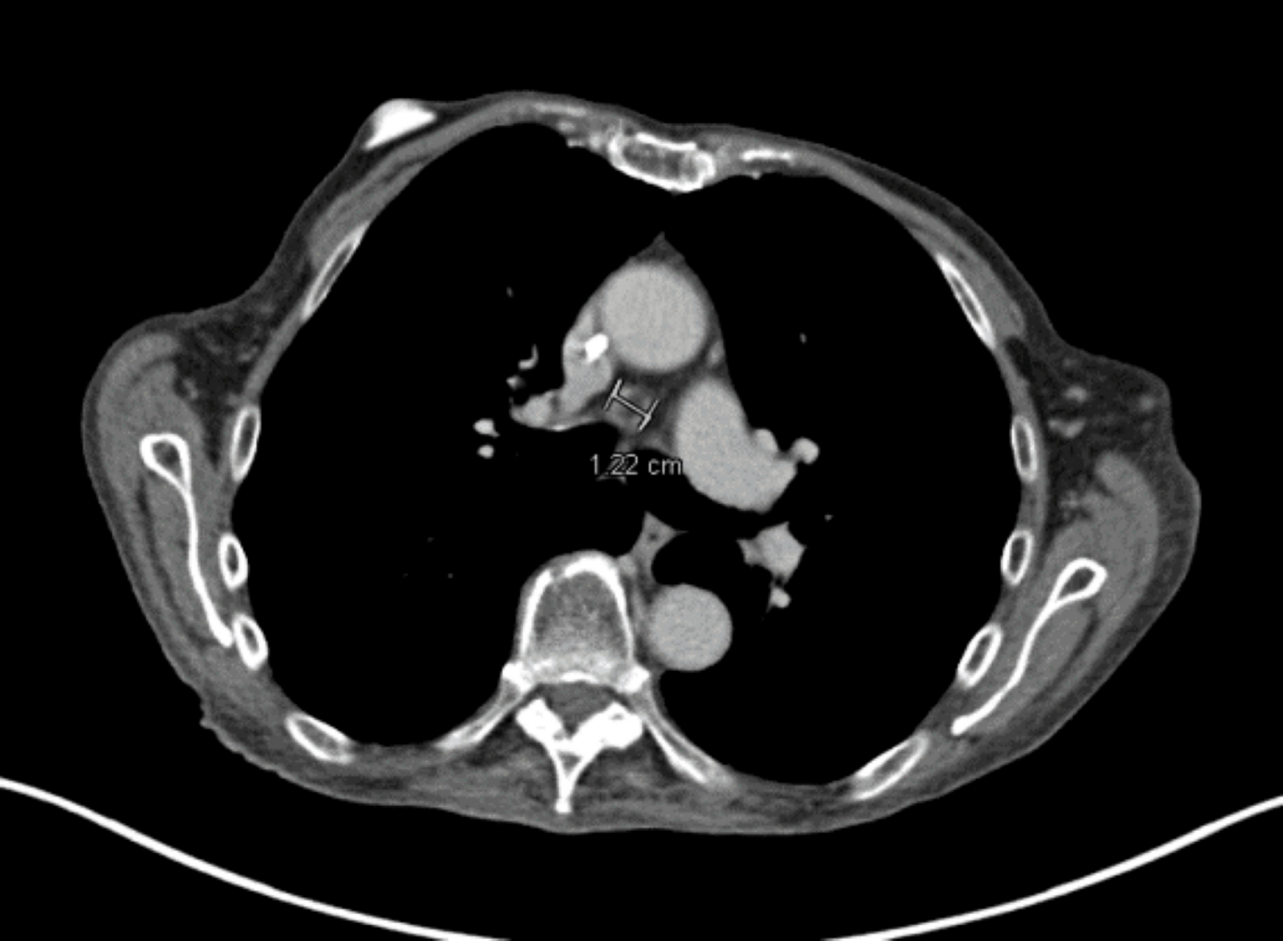
PET/CT scan at eight months after diagnosis Representative image from a repeat PET/CT at eight months, demonstrating a mediastinal lymph node, which had grown from previous imaging to 1.22 cm. Lymphadenopathy had also progressed in the epicardium, retroperitoneal area, and right supraclavicular region (not shown). CT, computed tomography; PET, positron emission tomography

An initial PET scan demonstrated lymphadenopathy in the left axilla, left neck, subcarina, and retroperitoneum (Figures 3A-3D). As he was asymptomatic and had a baseline poor performance status, we elected to withdraw immunosuppression alone without initiating chemotherapy about five months after diagnosis. EBV levels down-trended thereafter, with minor fluctuation over the next few months. Unfortunately, between months eight and nine after diagnosis, he developed new lymphadenopathy and up-trending EBV levels (Figure 4). A repeat CT/PET scan indicated progressive disease (Figure 5).

Given the rarity of this condition and specific subtypes, no standard chemotherapy protocol exists. Case series-level data suggest that using Hodgkin-inspired protocols can be effective [5]. However, he had a persistently low-performance status and was not a candidate for cytotoxic chemotherapy. As the clonal cells were positive for EBV, it was felt that he would benefit from rituximab therapy. Additionally, as the clonal cells were CD30-positive, brentuximab was added. Ten months after initial diagnosis, he was started on rituximab weekly at a dose of 375 mg/m^2^ with the appropriate premedication for six weeks total with concurrent brentuximab dosed at 1.2 mg/kg every three weeks for a total of two doses. This therapy was well-tolerated with no noted grade three toxicities by Common Terminology Criteria for Adverse Events (CTCAE) version 5.0. Imaging three months after treatment demonstrated a complete response.

**Figure 6:**
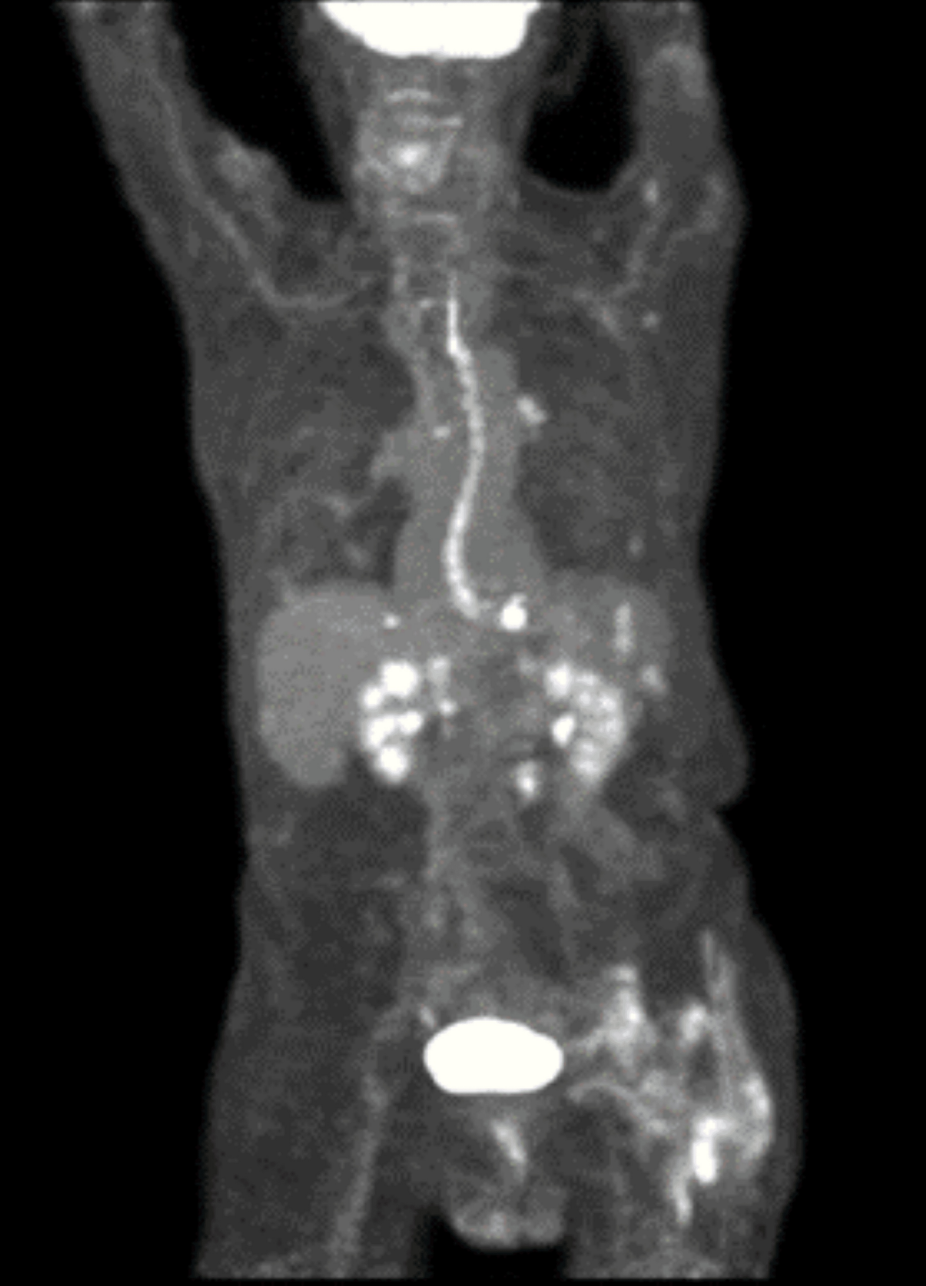
PET/CT between months 14-15 after diagnosis Full-body image demonstrating multiple areas of high FDG uptake, consistent with widespread lymphoproliferative disease. CT, computed tomography; PET, positron emission tomography; FDG, fluorodeoxyglucose

However, one month later, he presented with rapidly progressive B-symptoms that necessitated hospitalization. Repeat imaging demonstrated relapse (Figure 6). As his clinical situation continued to deteriorate, aggressive interventions were deferred, and he was discharged home to hospice. He expired a week later, 16 months after his initial diagnosis.

## Discussion

Several types of MTX-LPD have been identified. Among these are reactive lymphoid hyperplasia, polymorphic LPD, diffuse large B-cell lymphomas, classic Hodgkin lymphoma (CHL) [5], natural killer/T cell lymphoma, peripheral T cell lymphoma, and angioimmunoblastic T cell lymphoma [4]. The literature also describes a polymorphous B cell proliferation containing occasional Reed-Sternberg-like cells associated with MTX use, a disorder named "Hodgkin’s lymphoma-like LPD" in some reports [4]. This rare LPD was present in our patient. However, the pathologist reviewing our patient’s case termed his diagnosis “EBV-positive B-cell lymphoproliferative disorder with Hodgkin/Reed-Sternberg-like cells.” The rarity of this LPD subtype has made it difficult to elucidate its pathophysiology, much less the ideal approaches to its treatment, as will be discussed below. It should also be noted that MTX is associated with various solid organ malignancies and that concomitant use of biological therapies, such as adalimumab (as in this patient), further increases the risk of malignancy, specifically breast, ovarian, and lung cancers [6].

An important feature of all LPD is the presence or absence of EBV. EBV is a known oncogenic virus implicated in lymphomagenesis, particularly in the setting of immune deficiency. The rates of EBV positivity in LPD appear to be the highest in the post-transplant setting (63-95%) [7,8], and these data have provided the basis for exploring EBV positivity in other LPDs. One case series reported an EBV-positive rate of 27.1% among subjects with MTX-LPD. Although the association is not fully understood, it has been proposed that MTX directly induces reactivation of EBV infection. EBV then causes upregulation of oncogenes and other epigenetic changes in B cells, resulting in various clonal and polyclonal lymphoproliferation [9,10].

The management of iatrogenic LPD begins with discontinuation of the immunosuppressive drug. Importantly, it has been shown that treatment-related outcomes differ depending on the EBV status of the patient with LPD, so serologic and histopathologic identification of EBV positivity in our patient was critical. Specifically, many studies have demonstrated that the rate of spontaneous regression after MTX withdrawal is higher in EBV-positive compared to EBV-negative MTX-LPD [3,11]. For example, one study included nine RA patients who developed MTX-LPD (all non-Hodgkin lymphoma), and among the three with EBV infection, two obtained a complete response with MTX withdrawal alone, and one had stable disease after MTX withdrawal. The latter patient eventually required chemotherapy to achieve a complete response. In contrast, of the six patients who were EBV-negative, none obtained a complete response after MTX withdrawal; one obtained a partial response, one had stable disease, and four had progressive disease [12].

Similarly, Kamel et al. [13] published a report of eight patients with MTX-LPD, four with Hodgkin’s disease (HD) and four with lymphoproliferations resembling HD. Among those in the latter group, two experienced resolution of the lymphoid neoplasm with MTX discontinuation alone, and one obtained clinical response after MTX withdrawal and a course of glucocorticoid therapy. Two of the three with follow-up were EBV-positive. Among the four others with histopathologic features diagnostic of HD, three required either chemotherapy or radiotherapy to achieve a response. Only one patient in this group was positive for EBV. This case series was important both for its demonstration of the association between EBV status and response to MTX withdrawal, as well as its inclusion of lymphoproliferations (such as in our patient) with features resembling - but not diagnostic of - HD.

As highlighted above, not all cases of MTX-LPD respond adequately to the withdrawal of immunosuppression alone. Several second-line treatment options exist, including chemotherapy, monoclonal antibodies, and antivirals. The data to support these treatments are largely drawn from the post-transplant lymphoproliferative disorder (PTLD) literature. Notably, this consists mostly of a small number of case reports, retrospective studies, and very few prospective trials. The most robust of these data demonstrate at least some efficacy of the following therapies: Rituximab (an anti-CD20 monoclonal antibody) in the treatment of CD20-positive disease + sequential treatment with rituximab and CHOP (cyclophosphamide, doxorubicin, vincristine, prednisone) or R-CHOP chemotherapy; CHOP without rituximab in CD20-negative PTLD; antivirals; and immunomodulatory therapy (interferon (IFN)-alpha, interleukin (IL)-6 antibodies, intravenous immunoglobulin (IVIG), and EBV-specific T-cell therapy). Of note, this latter treatment strategy has typically been reserved for rare PTLD subtypes or relapsed/refractory disease [14]. Also notable is that, for EBV-positive LPD, rituximab monotherapy is a first-line treatment and has been shown to result in complete remission in more than 60% of patients [15]. Patients who fail rituximab have poor outcomes, and subsequent treatment options are limited [16].

As outlined in the case presentation, after the withdrawal of immunosuppression, our patient’s disease responded well with down-trending EBV levels. Later, however, surveillance testing revealed a rise in EBV levels and progressive lymphadenopathy. Our patient was ineligible for cytotoxic chemotherapy (i.e., CHOP) owing to his poor baseline functional status. Thus, the decision was made to pursue treatment with monoclonal antibodies - rituximab for his CD20- and EBV-positive disease, as well as brentuximab for his CD30-positive clonal cells. Brentuximab is an anti-CD30 monoclonal antibody frequently used in patients with Hodgkin lymphoma, anaplastic large-cell lymphoma, and other CD30-positive lymphomas [17]. The patient experienced a complete response to this combined immunotherapy. However, his brief period of remission was followed by an ultimately fatal relapse.

## Conclusions

In summary, our patient developed a rare LPD subtype from his long-term methotrexate use for RA, characterized by EBV-positivity and Hodgkin lymphoma-like features. He responded well initially to the withdrawal of immunosuppression alone but required a more aggressive intervention later on. He was not a candidate for chemotherapy, and thus we opted to treat his disease with a unique and less toxic immunotherapy protocol. To date, our case represents the first reported use of a combination of rituximab and brentuximab therapy to treat MTX-LPD. Not only was the treatment well-tolerated by the patient; but repeat imaging obtained shortly after treatment showed complete response, albeit short-lived. Therefore, in cases in which chemotherapy is contraindicated, treatment of such disorders with rituximab and brentuximab may be a viable option. Clinicians should be aware that the duration of the response to this therapy can be short, and cytotoxic therapies should strongly be considered if the patient’s performance and medical status permit.
